# P-891. Antimicrobial Utilization Trend in Small Community Hospitals Supported by a Collaborative, Consultative Network in the Southeastern United States

**DOI:** 10.1093/ofid/ofaf695.1099

**Published:** 2026-01-11

**Authors:** Tark Kim, Elizabeth Dodds Ashley, Jeannette Bouchard, April Dyer, Melissa D Johnson, Angelina Davis, Deverick J Anderson

**Affiliations:** Department of Internal Medicine, Soonchunhyang University Bucheon Hospital, Bucheon, Kyonggi-do, Republic of Korea; Duke Center for Antimicrobial Stewardship and Infection Prevention, Durham, NC; Duke Antimicrobial Stewardship Outreach Network, Elgin, SC; Duke Center for Antimicrobial Stewardship and Infection Prevention, Durham, NC; Duke University, Durham, North Carolina; Duke Center for Antimicrobial Stewardship and Infection Prevention, Durham, NC; Duke Center for Antimicrobial Stewardship and Infection Prevention, Durham, NC

## Abstract

**Background:**

Antimicrobial stewardship programs (ASPs) are essential to reduce antimicrobial resistance, but small hospitals often face challenges in securing ASP expertise. A collaborative network model may help overcome this limitation. We analyzed the impact of changes in ASP expertise and implementation on antimicrobial utilization (AU) trends in small community hospitals (less than 100 beds) supported by the Duke Antimicrobial Stewardship Outreach Network (DASON).

**Figure:**
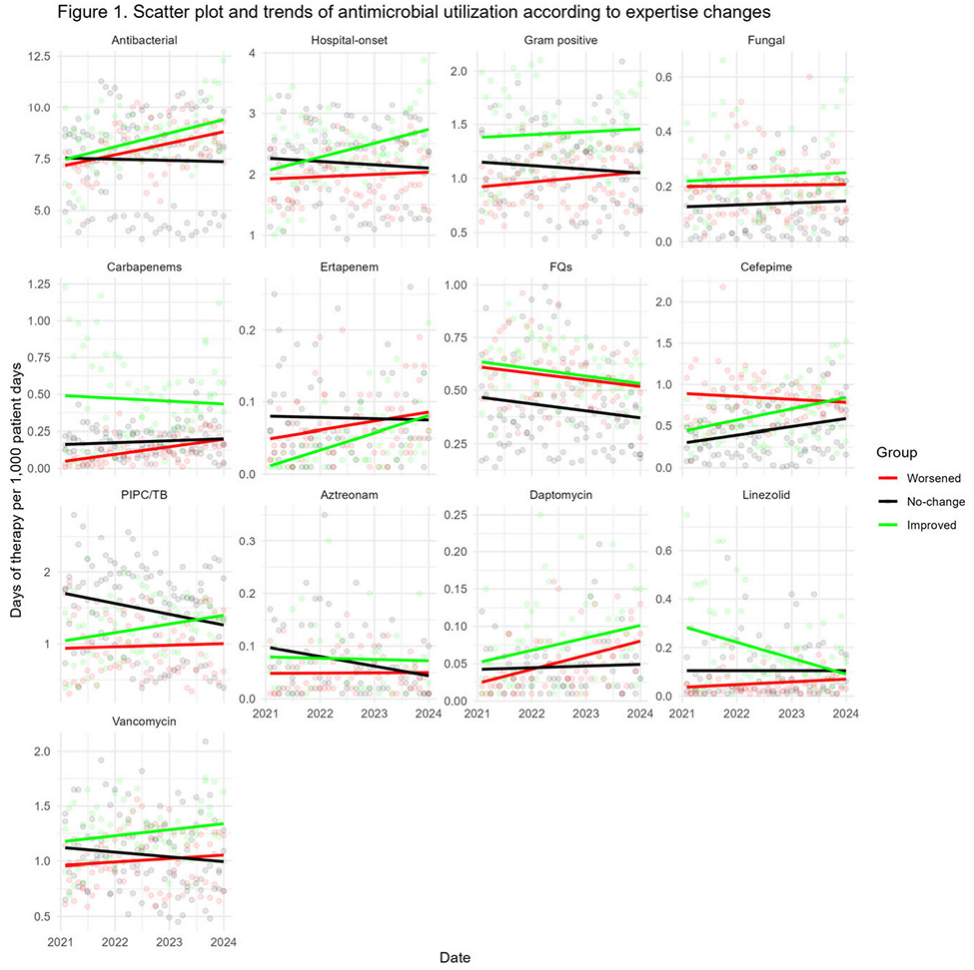

**Figure:**
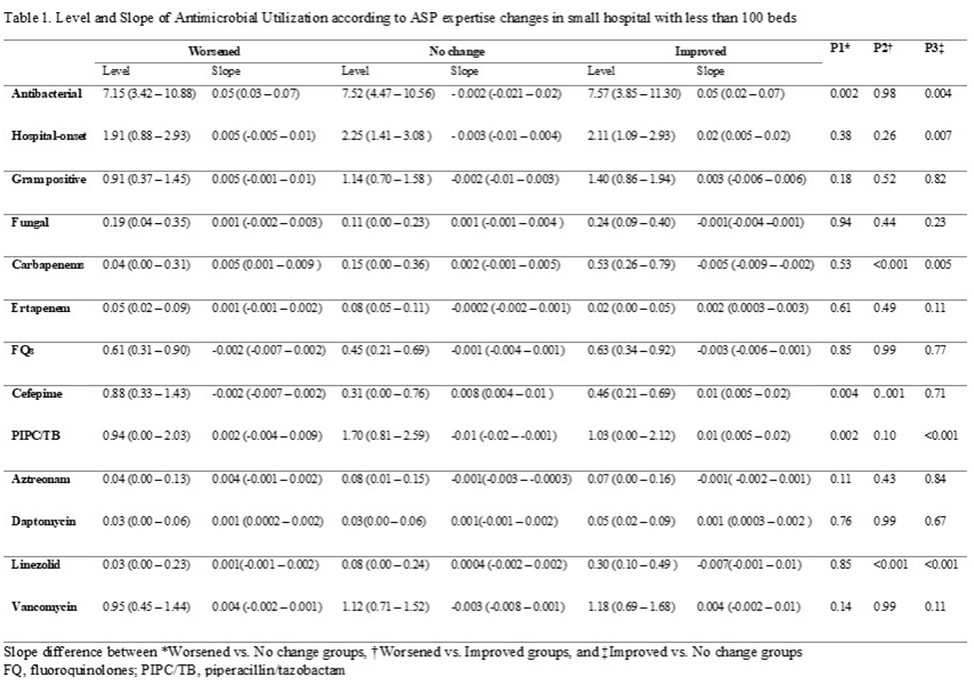

**Methods:**

We assessed the status of ASP expertise and implementation in hospitals with fewer than 100 beds who are members of the 48 hospital DASON network based on the 2021 and 2023 National Healthcare Safety Network (NHSN) annual surveys. AU data were obtained from the DASON database. Hospital were clustered into six groups in 2021 based on the composition of ASP expertise and implementation. Using the same clustering algorithm, hospitals were re-clustered in 2023. Within-hospital differences in implementation and expertise were categorized into worsened, no-change, and improved groups based on changes in their cluster assignments. A mixed-effects model was used to compare AU trends between groups, followed by post hoc analysis of slope changes from 2021 to 2023.

**Figure:**
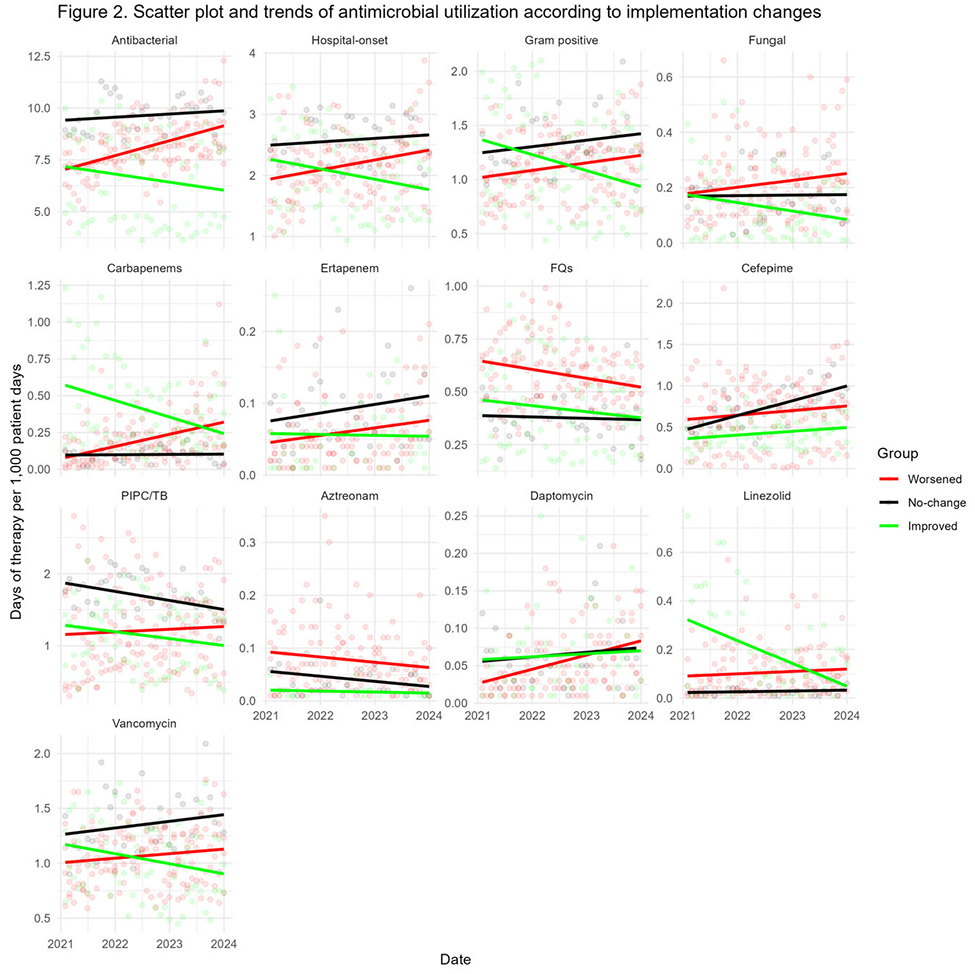

**Figure:**
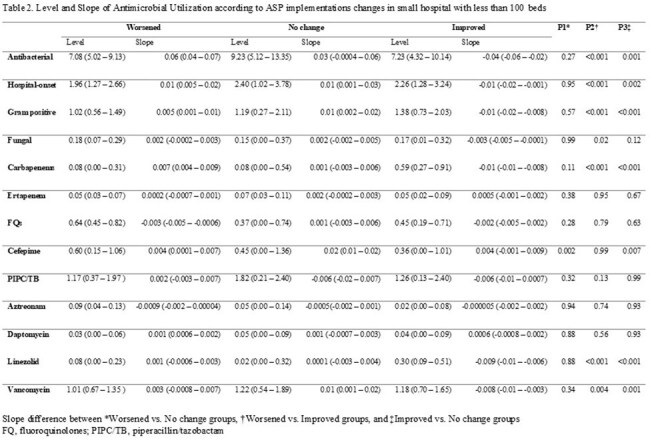

**Results:**

Of the 15 DASON member hospitals with fewer than 100 beds, 7 had complete AU and implementation and expertise data for analysis. Two had an infectious diseases (ID) physician, one had an ID pharmacist and six received ID tele-services in 2021. Regarding ASP expertise changes, three , two , and two hospitals were categorized into worsened, no-change, and improved groups, respectively. For ASP implementation, four, one and two hospitals were categorized into worsened, no-change, and improved groups. Linezolid and carbapenem use significantly decreased in the improved expertise group compared to the worsened and no-change groups. In the improved implementation group, AU for hospital-onset infections and Gram-positive organisms decreased significantly. Additionally, use of antifungal agents, carbapenems, linezolid, and vancomycin significantly decreased.

**Conclusion:**

Strengthening ASP implementation through a collaborative, consultative model may improve antimicrobial use even in small hospitals where securing expertise is challenging.

**Disclosures:**

Elizabeth Dodds Ashley, PharmD, MHS, HealthtrackRx: Advisor/Consultant|UpToDate, Inc.: Author Royalties Melissa D. Johnson, PharmD MHS AAHIVP, Biomeme: Licensed technology, method to detect fungal infection|Biomeme: Licensed technology, method to detect fungal infection|Scynexis: Grant/Research Support|Scynexis: Grant/Research Support|UpToDate: Author Royalties|UpToDate: Author Royalties

